# Evaluation of Cortical Bone Thickness of Posterior Implant Sites Using CBCT in Iraqi Population

**DOI:** 10.1155/2022/5723397

**Published:** 2022-09-05

**Authors:** Nuhad A Hassan, Aseel S. Khazaal Al-Jaboori, Afya Sahib Diab Al-Radha

**Affiliations:** ^1^Oral Medicine Department, College of Dentistry, Al-Mustansiriyah University, Baghdad, Iraq; ^2^Prosthodontics Department, College of Dentistry, Al-Mustansiriyah University, Baghdad, Iraq; ^3^Oral Surgery and Periodontology Department, College of Dentistry, Al-Mustansiriyah University, Baghdad, Iraq

## Abstract

**Background:**

Cortical bone thickness (CBT) is a critical factor for implant success and for determining the long-term dental implant treatment outcome.

**Objectives:**

The objective of this investigation was to examine posterior cortical bone thickness buccally and lingually in dentate and edentulous implant sites according to gender.

**Materials and Methods:**

CBT of 160 patients requiring a single posterior tooth implant was investigated by CBCT. The study included 80 males and 80 females. CBT was measured for implant edentulous sites at 3 levels including crestal bone (level 1), five mm from the crest (level 2), and ten mm from the crest (level 3). CBT was also measured for dentate sites at 3 levels including crestal bone (level 1), midroot bone (level 2), and apical portion (level 3). The differences of bone thickness between the levels of dentate sites were statistically analyzed using a Kruskal–Wallis one-way analysis of variance. Mann–Whitney test was used to determine the specific differences between group members. For the edentulous site, a one-way ANOVA was used.

**Results:**

CBT increased gradually from the crestal level to the apical level in all groups (buccal and lingual side, male and female). However, CBT at lingual side was statistically higher than that at buccal side in all groups. The mean value of CBT was significantly higher in males than females for both edentulous and dentate site. The dentate site shows a higher CBT in the apical level than the edentulous group in both male and female/buccal and lingual groups.

**Conclusion:**

CBT at the coronal levels is low and susceptible for resorption compared to the apical portion, especially for the female group. Moreover, CBT is thicker in males than females. It is essential to measure the CBT before making a treatment plan with dental implant prosthesis.

## 1. Introduction

Many factors cause the alveolar bone process in maxilla and mandible to undergo resorption such as teeth loss, age, osteoporosis, hormonal imbalance, metabolism disorder, and gender [1].

After tooth extraction and healing of the tooth socket, bone growth inside the tooth socket would be coordinated with alveolar ridge resorption. Bone loss would happen more obviously on the buccal aspect in horizontal and vertical direction [2], resulting in shorting and narrowing of the ridge [3].

Maxillary and mandibular bones are composed of apical part called basal bone and the alveolar bone which are formed by the alveolar process (the other part of the bones) [4]. Maxillary bone differs in structure from the mandibular that cancellous bone normally forms greater part of it [5].

In recent years, the basic method of treating edentulous regions is by dental implant, in which the lost tooth is replaced with dental implant to maintain the function and the esthetic for the patient. Different prosthodontic treatment is broadly employed in implants to replace single tooth or multiple teeth, to bear fixed prostheses, or to hold overdentures [6].

Implant success depends on osseointegration with the surrounding bone, which is influenced by the presence of enough amount of bone surrounding the implant [7, 8]. Therefore, volumetric bone assessment is essential for effective implantation surgery and for determining the success of implant treatment outcome. However, the quality of bone was determined by cancellous bone density inside and CBT outside; in addition to that, the ratio between them also has an effect [9]. Having poor bone quality could become a risk factor for implant failure because it might affect healing process and cause bone resorption [10].

To have a sufficient implant primary stability is considered the first requirement for the success of dental implant treatment, which depends on the mechanical interaction between dental implant fixture and the adjacent bone [11]. If there was insufficient primary stability, it could lead to micromotion during healing period and consequently may affect osseointegration, in which a fibrous tissue will be formed instead of bony tissue [12]. 150 *μ*m of micromotion is acceptable and considered a limit “gold standard,” so if an implant has micromotion lower than 150 *μ*m, this means it can be functionalized [13].

Having strong mechanical properties is important for primary stability; this mechanical engagement depends on many factors, one of which is related to bone. Consequently, bone thickness and density at the implant site affect osseointegration by having an effect on primary stability [14]. Therefore, there will be different types of healing according to the quantity and quality of the bone in the implant site. This has been pointed out by Farré-Pagés et al. who indicated in their study that increased bone quality has been linked with increased survival rate of dental implant [15]. In the same direction, Merheb et al. pointed out that primary stability can be anticipated by analyzing the characteristics of bone that the implant will be placed in [16].

Preoperative bone evaluation could prevent some complications such as perforation and injury to vital structures which can subsequently affect the success of dental implant. Moreover, satisfied alveolar bone volume, thickness, and height are important to achieve esthetically pleasing dental implant restorations [17, 18].

Recently, CBCT is regarded as the most efficient three-imaging modality for dental implants treatment. CBCT evaluation of alveolar ridge was recommended for implant placement after grafting procedures [19].

CBCT was introduced in 1982 and became important radiographic tool for diagnosis purposes and for giving good images without super imposition by close structures [20, 21]. It provided superiority to show anatomical structures compared to panoramic radiographs [21]. It supplies a very valuable information in all multiple planes compared to CT with a low radiation dose (15 times) and short time of scanning [21, 22].

CBCT is vital to analyze bone thickness prior to implant treatment. In a recent Iraqi study, CBCT was used preoperatively to ensure that the alveolar bone ridges were of satisfactory dimensions and density and to determine the correct length and width of the implant [23].

Miyamoto et al. in their research suggested that CBT has the strongest influence on the initial implant stability at the time of implant surgery and that the local bone condition (e.g., cortical and cancellous ratio) is essential for success of dental implant treatment [24]. Moreover, Di Stefano et al. emphasize in the conclusions of their review the importance of having a wider knowledge regarding the common values for CBT and the variable affecting CBT in order to choose the proper site for implant placement and select the accurate preparation methods [9].

A preoperative assessment of CBT before implant placement is important for long-term success of dental implant treatment [9]. Further clinical research regarding this needs to be undertaken. A lot of literature focused on analyzing bone resorption and bone thickness in anterior regions for esthetic. Therefore, the objective of this investigation was to (a) examine posterior cortical bone thickness buccally and lingually in dentate and edentulous sites prior to implant treatment and (b) identify if the gender had an effect on CBT.

## 2. Materials and Methods

This study involved 160 patients with partially edentulous sites divided equally according to gender [80 males (age range of 22–52 years) and 80 females (age range 22–47 years)]. The patients were referred to the Department of Oral Radiology/College of Dentistry/Al-Mustansiriyah University for dental treatment. Approval from the Scientific Committee of the Oral Medicine Department (2020.11.16) was obtained, and patient consent was gained for all subjects in accordance with the World Medical Association and the Declaration of Helsinki.

**Figure 1:**
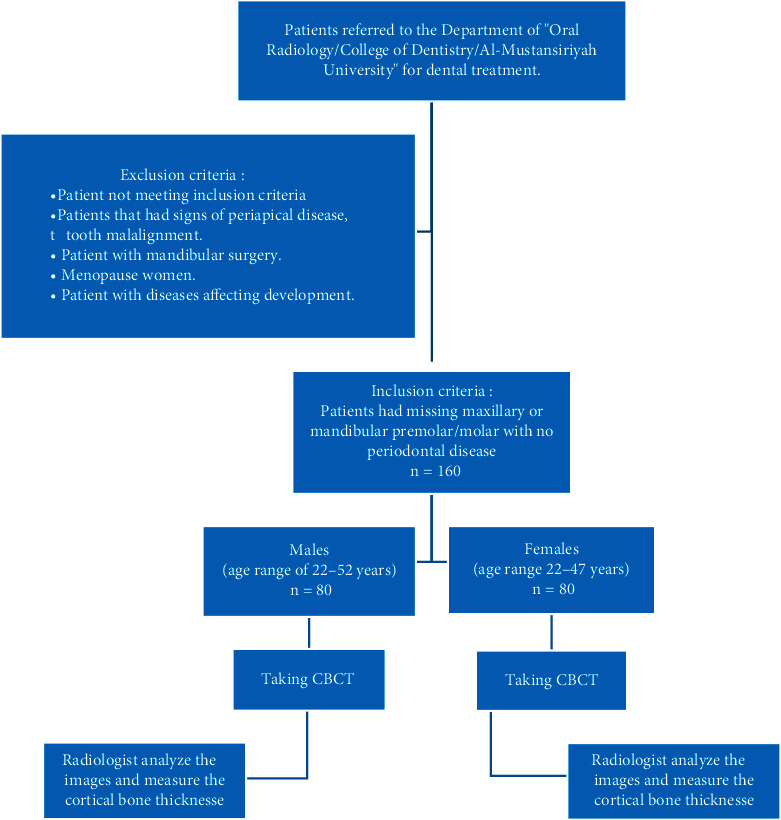
Flow diagram for the participants in the current study.

Inclusion criteria of this study included patients with missing maxillary or mandibular premolar/molar with no periodontal disease. While the cases of the exclusion criteria which were eliminated from the sample included patients with signs of periapical disease, mandibular surgery, tooth malalignment, and diseases affecting development and menopause women (Figure 1).

The sample size was measured using *G* power 3.1.9.7 (Program written by Franz-Faul, Universitatit Kiel, Germany). With power of study = 85% and alpha error of probability = 0.05, assume the Cohen's *D* effect size as 0.5 (medium). Under all these conditions, the sample size is 146 subjects adding 10% as error rate; thus, sample size will be 160 subjects (80 males and 80 females) {Effect sizes of Cohen's *D* are small = 0.2, medium = 0.5, and large = 0.8}.

Data were collected using Skyview: Myray, clefa dental group; Via Bicocca 14/*c*:40026 Imola (BO) Italy, scan time (sec):16.2, exposure time (sec): 8.1, energy (kv): 90; voxel size (mm): 0.3 × 0.3 × 0.3.

### 2.1. Image Evaluation

All measurements were estimated and renewed after 7 days by the same radiologist to get the mean of each value. To gain less artifacts by metallic filling which may cause beam hardening or streaking, these teeth put in place away from the center of FOV [25].

**Figure 2:**
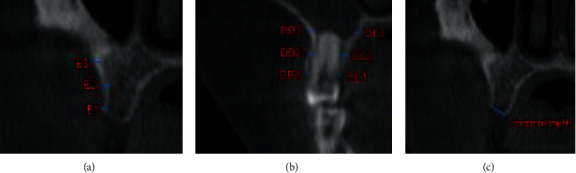
(a) Horizontal line (H) drawing at bone ridge level from the buccal to the palate wall. (b) Alveolar bone thickness at dentate sites in the coronal plane, measured perpendicular to three different levels of the long axis of the root on buccal and lingual walls, respectively: crestal bone level (DB1, DL1), mid root level (DB2, DL2), and apical bone level (DB3, DL3). (c) Cortical bone thickness measured for edentulous sites at the three levels: crestal bone thickness (E1), bone thickness 5 mm from E1 (E2), and bone thickness 10 mm from E1 (E3). ^#^ DB: dentate for buccal side and DL: dentate for lingual side.

The teeth located directly on the mesial and distal sides of the edentulous site were examined on the image. Therefore, if the first molar had been lost, the second premolar and molar were investigated for bone thickness. A line was drawn vertically along the long axis of the target tooth through sagittal plane as a reference and another line was drawn horizontally from the buccal to the lingual surface at the place of the ridges [Figure 2(a)]. In coronal plane, cortical bone thickness of the selected tooth was measured on buccal and lingual sides along the long axis of the root vertically at 3 various levels [Figure 2(b)] including the following:

- Level 1: dentate, crestal bone thickness (DB1 for buccal and DL1 for lingual)
- Level 2: dentate, mid root bone thickness (DB2 for buccal and DL1 for lingual)
- Level 3: dentate, apical portion of the tooth bone thickness (DB3 for buccal and DL3 for lingual)

At the center of the edentulous area mesiodistally, cortical bone thickness was measured at three levels (Figure 2(c)) [26]:

- Level 1: edentulous crestal bone thickness (E1)
- Level 2: edentulous bone thickness 5 mm from E1 (E2)
- Level 3: edentulous bone thickness 10 mm from E1 (E3)

Statistical analysis was carried out using Excel and IBM SPSS Statistics 24 programs.

Methods used to analyze and assess the results were descriptive statistics: mean, standard error, and medians.

The differences of bone thickness between the levels of dentate sites were statistically analyzed using a Kruskal-Wallis one-way analysis of variance. Mann–Whitney test was used to determine the specific differences between the group members. For edentulous site, a one-way ANOVA was used.

The independent sample *t*-test was used to test the differences between female and male groups and between buccal and lingual sides at each level. A three-way ANOVA was used to determine the effect of independent variables such as levels, sex, and site simultaneously on the dependent variable (bone thickness).

The probability value (*P*-value) is considered significant at *P* < 0.05 and highly significant if *P* < 0.01.

## 3. Results and Discussion

### 3.1. Results

Bone thickness of 160 patients requiring a single posterior tooth implant was investigated. The study included 80 males (age range of 22–52 years) and 80 females (age range 22–47 years). Cortical bone thickness was measured at 3 levels of mesial and distal located dentate sites and edentulous sites.

**Figure 3:**
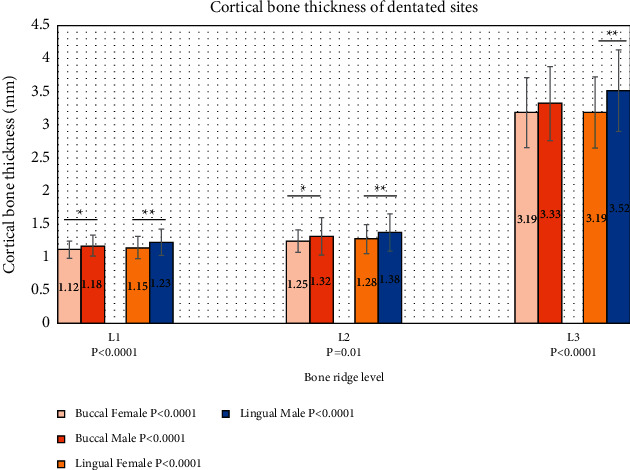
Cortical bone thickness of dentate site according to levels in female (*n* = 80) and male (*n* = 80) groups. *P* values in the legend represent the significance between bone thickness at 3 levels in the same site and gender group. *P* values in *X* axis represent the significance of bone thickness in the group level. (^*∗*^) Significant difference. (^*∗∗*^) Highly significant difference.

Figure 3 illustrates increase in bone thickness for the dentate sites from the crest (level 1) to the apical portion (level 3) at each buccal and lingual side in female and male patients.

However, a uniform picture of steady increase in CBT from the crestal level to the apical level can be seen in female dentate groups, in which the lowest value at the buccal side was in level 1 (1.12 ± 0.13 mm); then, it increases steadily to reach its highest value at level 3 (3.19 ± 0.53 mm). Similar picture can be seen in female dentate groups at lingual side. There were no significant differences between buccal and lingual sides within each group. However, a high significant difference was detected among the groups (*P* < 0.0001, Kruskal-Wallis test).

The mean CBT for the male group buccally was 1.18 ± 0.16 mm at level 1, which increased to 3.33 ± 0.56 mm at level 3. Similarly, CBT was increased for male/lingual groups from the crest to the apical portion (1.23 ± 0.20 mm to 3.52 ± 0.62 mm) with no significant difference between buccal and lingual sides within each group except for the apical root group as there were highly significant differences between buccal and lingual CBT with this group (*P*=0.04). Cortical bone thickness was significantly different between the levels on buccal and lingual sides (*P* < 0.0001, Kruskal-Wallis test), as shown in Figure 3.

Regarding the differences between females and males in the dentate sites, the mean of buccal CBT was significantly larger in males than females at crestal and mid root levels (levels 1 and 2, respectively) (*P* < 0.05). Meanwhile, the lingual bone showed statistically significant difference between males and females at all group levelswith *P* = 0.01, 0.02, and < 0.0001 for crestal, mid root, and apical root CBT, respectively. The CBT mean values for male groups were consistently higher than those for female groups.

**Figure 4:**
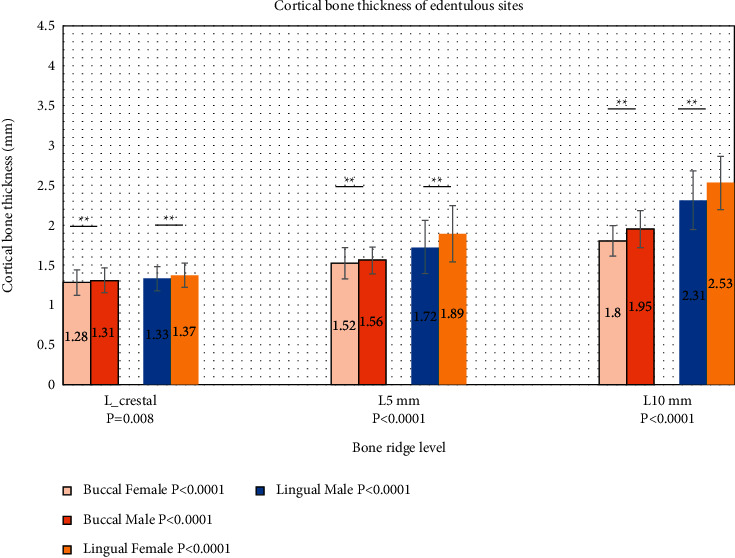
Cortical bone thickness of edentulous site according to levels in female (*n* = 80) and male (*n* = 80) groups. *P* values in the legend represent the significance of bone thickness at 3 levels in the same site and gender group. *P* values in *X* axis represent the significance of bone thickness in the group level. (^*∗*^) Significant difference. (^*∗∗*^) Highly significant difference.

In case of CBT measured on the edentulous sites, similar to dentate sites, CBT was gradually increased from level 1 (crestal bone) to level 3 in both gender groups. In females, crestal bone thickness was recorded as 1.28 ± 0.160 mm and increased to reach 1.8 ± 0.191 mm at 10 m length. The same picture was seen in male group with slightly higher CBT in which it started at 1.31 ± 156 at crestal bone to reach 1.95 ± 0.229 at 10 m length. However, the differences in mean values were significant across the 3 levels as indicated by *P* < 0.0001 in both sexes (Figure 4). Similar picture can be seen at lingual side.

When comparing the sexes at each level in the edentulous sites, the mean of the CBT levels was statistically higher in males than females at all levels (*P* < 0.0001, *t*-test analysis).

An interesting result can be seen in Table 1, as the dentate site shows a higher cortical bone thickness in level three than the edentulous group in all groups (buccal and lingual side, male and female). The opposite picture can be seen for both level 1 and level 2 as the CBT in edentulous group was higher than that in dentate group in all groups.

This study was also interested in whether the thickness of bone for the dentate sites was influenced by specific factors: level of measurements, sex, and site of measurement (buccal or lingual). Therefore, three-way ANOVA was conducted to study the association between these factors (Table 2).

The effects of levels and sex on bone thickness were significant (*P* < 0.0001). Site effect was also statistically significant (*P* < 0.001).

The interaction effect between the factor of bone levels and sex was statistically significant (*P*=0.024) and was insignificant between levels and site (*P*=0.58). Meanwhile, the interaction between sex and site was significant with *P*=0.027. Additionally, the effect of interaction between the three factors was statistically insignificant with *P*=0.301.

### 3.2. Discussion

Alveolar process of the jaw bone developed concurrently with the teeth eruption. Thus, bone resorption after tooth extraction is inevitable alteration that will in turn affect oral health and function [27, 28]. In addition to that, endodontic lesions and trauma might cause an earlier bone loss that would have an impact on bone resorption after teeth loss [29]. Even with the presence of teeth, periodontal diseases can cause bone resorption by bacterial action, which can be subjected to detoxification by using mouth rinses [30]. Such situations will make constructing dentures or implant supported prosthesis pose a challenge to the dental team to overcome the negative effects of bone resorption.

However, bone resorption could occur after placing dental implant, as the peri-implant mucosities and consequently peri-implantitis cause bone resorption and may lead to implant failure [31]. For this reason, Butera et al. suggested using specific periodontal treatment gel as a long period therapy for peri-implant tissue and recommended further study regarding this [32].

The presence of favorable architecture of the alveolar ridge and a sufficient alveolar bone volume is essential to obtain better functional and esthetic prosthetic restorations. Moreover, having a thick CBT is connected to increased dental implant stability and subsequently increased competency for prosthesis load bearing [33].

Therefore, evaluation of bone prior to tooth replacement with dental implant by using a well-advanced technique such as CBCT is essential for better clinical outcomes [20].

CBCT is valuable in preoperative evaluation of implant measurements, and it can be easily manipulated for improving the esthetic and functional outcome and for avoiding complications. Furthermore, it is an accessible tool for following up after implant surgery [34].

In a previous study, Padhye and Bhatavadekar [35] investigated 349 dentulous sites from 250 CBCTs to assess alveolar bone (bucco-palatal thickness) before implant surgery of posterior maxilla, and they suggested a vital role of CBCT for good diagnosis.

In the present study, CBT of posterior dentate sites was measured and recorded buccally and lingually across the entire root length. Bone width was measured at various levels (crest, mid root, and apical portion of the tooth), and it was more than 1 mm at both buccal and lingual sides of the teeth at all 3 recorded levels, which verified the requirement of Oettlé et al. (2015) [36] as they deduced in their study that abundant bone thickness (at least 1 mm) throughout the implant is the target for implant success.

Kolte et al. [26] in 2020 carried out a study involving 100 patients by CBCT and concluded that bone thickness was steadily increased from the crest to the apical portion for both lingual and buccal sides with a high statistically significant difference between the levels which is in accordance with this study. Additionally, they recorded a significant effect of sex on bone width on buccal and lingual sides of the dentate sites at all the levels and at level 1 of the edentulous sites.

The current study revealed that males significantly showed a higher CBT than females in the buccal side at crestal and mid root levels (level 1 and 2) and at all levels lingually for dentate sites, indicating the high influence of gender which was also determined through the three-way ANOVA.

For the edentulous sites, bone thickness was significantly larger in males than females at all levels, and this is in agreement with Koç and Erdem [37] who investigated alveolar crest width in subjects with edentulous mandible by CBCT of 38 females and 39 males and reported that males showed higher statistically significant crestal width than females (*P*=0.039) which is in conformity with the findings of the present study.

However, many researchers have pointed that the bulk of bone in males is higher than females. Fayed et al. [38] evaluated crest width on CBCT images and noticed that males had significantly larger measures than females of dentate mandible through a study of mandibular crest width of 100 dentate patients (13–27 years old including 54 females and 46 males). In their study, the cortical thicknesses were significantly higher in males buccally and palatally in both upper and lower jaws but at specific sites and levels, which is compatible with the current study. Moreover, the present study is in accordance with the findings of Zhang et al. [39] who examined alveolar crests of 28 males and 31 females and realized that females and edentulous regions had smaller alveolar thickness than males and dentate regions, respectively. Thickness of buccal bone decreased from apical to coronal in edentulous and dentate regions.

The mean CBT values at the edentulous sites were higher in males when compared to females at the most coronal levels, in accordance with Kolte et al. [26] who found this difference only at the alveolar crest. The differences in bone width were found to be statistically significant at the three levels for both sex groups. The present study emphasized that bone width measurements were done in the center of the edentulous site mesiodistally, which is more exposed for bone resorption after tooth extraction.

The current study was also in line with Cassetta et al. [40] who reported a decrease of bone thickness from base to crest of alveolar bone, and the alveolar cortical bone thickness in maxilla was smaller in females than in males.

This study also showed an interesting finding regarding the feature of minimal difference of bone thickness between buccal and lingual bone at all levels for male group and at levels 1 and 2 for female group. This difference could be regarded as one of the causes of the low rate of resorption of the lingual sides after teeth extraction. This result was in partial agreement with the finding of previous authors which revealed statistically significant in all levels for both gender groups between buccal and lingual bones. This difference in results could be related to the difference in sample population [26, 41]. The authors attributed this result to the pronounced lingual inclinations of the alveolar process. Therefore, implant angulation and placing the implant at an ideal prosthesis-driven location become essential to guarantee proper distribution of shear forces on the jaw bone [42]. However, one of the difficulties that face the surgeon during implant surgery is the tendency of the drill to drift buccally [43], and as in the results of current research, this drifting could be attributed to the differences in CBT between the buccal and lingual plate which shed the light on the importance of estimating such CBT before staring implant treatment in order to give the surgeon a clue to expect such a drifting during surgery.

A remarkable result is that CBT is higher in edentulous bone than in dentate bone for the first two levels, which disagree with Katranji and coworker [44]. This could be due to the fact that the alveolar bone starts to resorb immediately after tooth extraction [45]; this hypothetically makes the middle root in the dentate area equal to the crestal level in edentulous site.

In the lowest level (L 3/*L* 10 m), the CBT in dentate site was higher than in the edentulous area in both male and female groups (also buccal and lingual). This could be attributed to the maximum force of mastication transmitted to the apical portion of the tooth giving the functional bone to be thick enough to withstand such a force. However, once the bone loses such stimulation (after tooth extraction), it will decline and start to lose its thickness [46]. This was also pointed out by Schwartz-Dabney and Dechow [47] who stated in their research that extraction of a tooth will not cause only resorption of the ridge but it will also cause many “microstructural changes” in cortical bone in which one of these changes is CBT.

Certain limitations are found in the current investigation. It combined the dimensions of maxillary and mandibular sites. Moreover, edentulous duration and age were not seen as determining factors that can affect bone dimensional variations. Adding to this, in this research, patient biological factors such as calcium level and vitamin D level are not considered.

Future research direction would be to measure the CBT before implant placement followed by evaluation of implant stability during the healing period to find out the impact of CBT on dental implant healing and success rate.

## 4. Conclusions

Within the limitations of this study, CBT in the dentate sites was low and susceptible for resorption at the coronal level compared to the apical portion especially for the female group. CBT is thicker in males than females indicating that the surgeon should be more careful during placing implant in females not to perforate the cortical bone specially buccally. Furthermore, it is essential to measure the CBT before making the decision of performing immediate dental implant prosthesis.

## Figures and Tables

**Table 1:** Cortical bone thickness in millimeter (mm) of dentate and edentulous sites according to levels in female and male groups (*n* = 80).

	Cortical bone thickness of dentate sites and edentulous sites							
	Buccal				Lingual			
	Female **dentate** (mm)	Female **edentulous** (mm)	Male **dentate** (mm)	Male **edentulous** (mm)	Female **dentate** (mm)	Female **edentulous** (mm)	Male **dentate** (mm)	Male **edentulous** (mm)
L 1/L_crestal	**1.12** **±** **0.13**	**1.28** **±** **0.160**	**1.18** **±** **0.16**	**1.31** **±** **0.156**	**1.15** **±** **0.17**	**1.33** **±** **0.152**	**1.23** **±** **0.20**	**1.370** **±** **0.152**

L 2/*L* 5 mm	**1.25** **±** **0.17**	**1.52** **±** **0.198**	**1.32** **±** **0.29**	**1.56** **±** **0.167**	**1.28** **±** **0.22**	**1.72** **±** **0.333**	**1.38** **±** **0.28**	**1.89** **±** **0.353**

L 3/*L* 10 m	**3.19** **±** **0.53**	**1.8** **±** **191**	**3.33** **±** **0.56**	**1.95** **±** **0.229**	**3.19** **±** **0.54**	**2.31** **±** **0.372**	**3.52** **±** **0.62**	**2.53** **±** **0.328**

**Table 2:** Three-way ANOVA test of between-subject effects {level of measurements, sex, and site of measurement (buccal or lingual)}.

Source	Type III sum of squares	Df^a^	Mean square	F^b^	Sig.^c^
Corrected model	927.759	15	61.851	372.393	0.000
Intercept	4667.887	1	4667.887	28104.637	0.000
Levels	915.844	3	305.281	1838.052	0.000
Sex	6.844	1	6.844	41.210	0.000
Site	1.751	1	1.751	10.542	0.001
Levels ^*∗*^ sex	1.571	3	0.524	3.154	0.024
Levels ^*∗*^ site	.323	3	0.108	0.649	0.584
Sex ^*∗*^ site	.817	1	0.817	4.920	0.027
Levels ^*∗*^ sex ^*∗*^ site	.608	3	0.203	1.221	0.301
Error	209.937	1264	0.166	—	—
Total	5805.583	1280	—	—	—
Corrected total	1137.696	1279	—	—	—

Df^a^: degree of freedom, F^b^: F-statistics, and Sig^c^: significant.

## Data Availability

The data used to support the findings of this study are included within the article.
